# Accuracy of Rapid IgM-Based Immunochromatographic and Immunoblot Assays for Diagnosis of Acute Scrub Typhus and Murine Typhus Infections in Laos

**DOI:** 10.4269/ajtmh.2010.09-0534

**Published:** 2010-08-05

**Authors:** Stuart D. Blacksell, Kemajittra Jenjaroen, Rattanaphone Phetsouvanh, Ampai Tanganuchitcharnchai, Phonlavanh Phouminh, Simalee Phongmany, Nicholas P. J. Day, Paul N. Newton

**Affiliations:** Wellcome Trust-Mahosot Hospital-Oxford Tropical Medicine Research Collaboration, Microbiology Laboratory, Mahosot Hospital, Vientiane, Laos; Centre for Tropical Medicine, University of Oxford, Churchill Hospital, Oxford, United Kingdom; Mahidol-Oxford Tropical Medicine Research Unit, Faculty of Tropical Medicine, Mahidol University, Bangkok, Thailand

## Abstract

We studied the diagnostic accuracy of a rapid immunochromatographic test (ICT) for detection of IgM against scrub typhus (ST ICT) and an immunoblot test for the detection of IgM against murine typhus (MT IBT) by using admission serum samples from 1,030 febrile patients in Laos. Sensitivity and specificity for the ST ICT determined by using the diagnostic criteria of a four-fold increase in IgM against *Orientia tsutsugamushi* between paired samples were 23.8% (95% confidence interval [CI] = 15.9–33.3%) and 86.2% (95% CI = 84.1–88.6%), respectively. Sensitivity and specificity for the ST ICT determined by using an admission IgM titer ≥ 1:400 were 39.1% (95% CI = 34.1–44.2%) and 99.5% (95% CI = 98.7–99.9%), respectively. Sensitivity and specificity for the MT IBT determined by using the criteria of a four-fold increase in IgM against *Rickettsia typhi* between paired serum samples were 61.2% (95% CI = 53.7–68.3%) and 86.5% (95% CI = 84.1–88.8%), respectively. Sensitivity and specificity for the MT IBT determined by using an admission IgM titer ≥ 1:400 were 54.6% (95% CI = 49.1–60.0%) and 94.1% (95% CI = 92.0–95.7%), respectively. Both assays had relatively good specificity but low sensitivity and thus have limited utility for admission diagnosis.

## Introduction

Scrub typhus, caused by *Orientia tsutsugamushi*, and murine typhus, caused by *Rickettsia typhi*, are important acute febrile illnesses in the Asia–Pacific region.^1^–^4^ Clinical diagnosis of typhus is difficult because signs and symptoms are similar to those of many other febrile diseases such as typhoid, leptospirosis, and dengue virus infection. Laboratory diagnosis of scrub typhus and murine typhus infection has relied on the detection of antibodies to *O. tsutsugamushi* and *R. typhi*, and the gold standard assay is the indirect immunofluorescent antibody (IFA) assay.^5^ Development of rapid diagnostic tests using immunochromatographic test (ICT) or immunoblot test (IBT) technologies has provided a mechanism for point-of-care serologic testing. To date, there is limited information on the utility of rapid tests for the diagnosis of acute scrub and murine typhus, and published information has concentrated on whole antibody rather than IgM-specific tests.^6^

We assessed the diagnostic capacity of a commercially available rapid ICT for detection of IgM against *O. tsutsugamushi* and an IBT for detection of IgM against *R. typhi* to aid the diagnosis of acute scrub and murine typhus infection in patients in the tropical and disease-endemic environment of the Lao People's Democratic Republic (Laos).

## Materials and Methods

### Patient samples.

The study was conducted at Mahosot Hospital in Vientiane, Laos during March 2003–May 2007. Ethical clearance was granted by the Ethical Review Committee of the Faculty of Medical Sciences, National University of Laos, Vientiane, Laos, and by the Oxford University Tropical Ethics Research Committee, United Kingdom. After informed written consent was obtained, consecutive inpatients of any age were recruited if the responsible physician suspected typhus, characterized by a minimum of fever, headache, and/or myalgia. Venous blood samples were collected on the day of admission and during convalescence at or after discharge from the hospital. Serum was divided for immediate use and for storage at –80°C.

### Indirect immunofluorescent antibody assay.

IgM against scrub typhus (*O. tsutsugamushi* pooled Karp, Kato, and Gilliam antigens) and murine typhus (*R. typhi* Wilmington strain antigen) IgM was detected by using an IFA assay.^5^ Slides for the IFA assay were obtained from the Australian Rickettsial Reference Laboratory (Geelong, Victoria, Australia). Briefly, patient serum samples was serially diluted two-fold from 1:100 to 1:25,600 in phosphate-buffered saline (PBS) containing 2% (w/v) skim milk powder, incubated in a humidified atmosphere for 30 minutes at 37°C, and washed three times in PBS. Anti-human IgM fluorescein isothiocyanate conjugate (Jackson ImmunoResearch Laboratories, West Grove, PA) diluted in PBS–skim milk powder diluent containing 0.00125% (w/v) Evans Blue counterstain was applied to all wells, and wells were incubated in a humidified atmosphere for 30 minutes at 37°C. Slides were examined by epifluorescence microscopy (BX50; Olympus, Tokyo, Japan) by two observers at a magnification of 400×. The binding endpoint titer was determined as the highest titer that showed fluorescence.

### Scrub typhus immunochromatographic test.

The scrub typhus ICT (Panbio, Sinnamon Park, Queensland, Australia) (ST ICT) was performed on admission-phase specimens according to the manufacturer's instructions. Briefly, 5 µL of serum was applied to the reagent pad of the ICT strip and two drops of buffer was added. Results were read visually 10 minutes later. Results were recorded as positive, equivocal, or negative for the IgM against *O. tsutsugamushi* and control lines. Because the tests were performed in a routine hospital laboratory with staff rotation, these tests were read individually by trained operators under the direction of the study supervisor at Mahosot Hospital.

### Murine typhus Dip-S-Ticks test.

The Murine typhus Dip-S-Ticks IBT (Panbio) (MT IBT) was performed on admission specimens according to the manufacturer's instructions with the modification that the manufacturer provided goat anti-human IgG and alkaline phosphatase–conjugated goat anti-human IgM to make the assay specific for detection of IgM. Samples were assessed by trained staff in a routine hospital laboratory, as described above. Using the interpretation provided by the manufacturer, the presence of ≤ 2 dots was considered not seroreactive with IgM against *R. typhi* and thus negative. Samples that resulted in 3 or 4 dots were considered seroreactive with IgM against *R. typhi*.

### Data analysis.

Diagnostic accuracy was calculated using ST ICT and MT IBT results compared with the gold standard reference IFA assay results for each patient sample. The gold standard reference IFA assay result used in this study was that an admission-phase sample IgM IFA assay titer ≥ 1:400^7^ or a four-fold increase between paired admission-phase and convalescent-phase serum samples was diagnostic for scrub typhus. In this study, we have also used a combination of these diagnostic criteria (i.e., combined ≥ 4-fold increase in titer or admission-phase titer ≥ 1:400) as a reference comparator. We also used these criteria for serologic diagnosis of murine typhus infection because there are no evidence-based guidelines for IFA assay cut-off values for this disease. Equivocal ST ICT and MT IBT results were considered negative for the final analysis. A 2 × 2 table was constructed where the gold standard result was cross-tabulated with the ST ICT and MT IBT results to define true-positive, false-positive, false-negative, and true-negative values. Standard diagnostic accuracy indices of sensitivity, specificity with exact 95% confidence intervals (CIs) and interquartile ranges (IQRs) of days of fever were calculated by using Stata/SE 8.0 (Stata Corp., College Station, TX). Significant differences (*P* < 0.05) between rapid test positivity rates and days of fever and IgM IFA assay titer and assay cross-reactivity using different diagnostic criteria were calculated by using Pearson's chi-square test.

### Assessment of diagnostic utility.

To examine the true diagnostic utility of the rapid tests in a clinical setting, four questions were posed.

1) In a patient with suspected acute typhus infection, how accurate were the ST ICT and MT IBT for diagnosis of scrub and murine typhus, respectively, in absolute terms when compared with the above mentioned established IFA diagnostic criteria?^7^ This comparison rates the ability of the test to make the correct diagnosis on the admission-phase sample compared with the final, retrospective, gold standard diagnosis made using admission-phase and convalescence-phase serum samples. It is a test of the accuracy of the rapid tests because there may not be sufficient IgM present on admission in a patient ultimately diagnosed as having the disease on the basis of increasing titers with a paired convalescent-phase sample. Thus, this tests the premise on which the rapid diagnostic test is based and the ability of the test to correctly detect the presence of IgM.

2) What is the relative accuracy of the admission ST ICT and MT IBT for the detection of IgM compared with the gold standard *O. tsutsugamushi* and *R. typhi* admission-phase IgM IFA assays?

3) What is the effect of cross-reactivity between antibodies to *O. tsutsugamushi* and *R. typhi* on the diagnostic utility of ST ICT and MT IBT compared with gold standard IFA assays for antibodies against *O. tsutsugamushi* and *R. typhi*?

4) What is the effect of sample timing, in relation to the duration of illness, on the ST ICT and MT IBT positivity rates for IgM against *O. tsutsugamushi* and *R. typhi* IFA assay-positive patients?

## Results

### Patient samples.

Admission-phase and convalescent-phase serum samples (n = 2,060) from 1,030 patients (median age = 28 years, IQR = 20–42 years) were evaluated by *O. tsutsugamushi* and *R. typhi* IgM IFA reference serologic analysis to determine scrub and murine typhus antibody status of the patients. A total of 8.3% (86 of 1,030) of the patients were children (age < 15 years). All patients were assessed by using the ST ICT and the MT IBT. Median number of days of fever at the time of collection of the admission-phase sample was 6 (IQR = 5–10 days), and median interval between obtaining admission-phase and convalescent-phase samples was 7 days (IQR = 5–10 days).

### Reference assay.

For scrub typhus IFA reference testing, 101 (9.8%) of 1,030 patients had a ≥ 4-fold increase in titer between paired specimens in *O. tsutsugamushi* IFAs, and 376 (36.1%) of 1,030 patients had admission-phase IgM titers ≥ 1:400. Four hundred twenty-six (41.4%) of 1,030 patients had admission-phase IgM titers ≥ 1:400 or a ≥ 4-fold increase in titer between paired specimens (Table 1).

For murine typhus IFA reference testing, 183 (17.8%) of 1,030 patients had a ≥ 4-fold increase in titer between paired specimens in the *R. typhi* IFAs, and 339 (32.9%) of 1,030 patients had admission-phase IgM titers ≥ 1:400. Four hundred nine (39.7%) of 1,030 patients had admission-phase IgM titers ≥ 1:400 or a ≥ 4-fold increase in titer between paired specimens (Table 1).

### Diagnostic accuracy questions.

1) In a patient with suspected acute typhus infection, how accurate were the ST ICT and MT IBT in diagnosis of scrub and murine typhus, respectively, in absolute terms, when compared with previously established diagnostic criteria for scrub typhus?

For the ST ICT, when we used the stringent ≥ 4-fold increase in IFA titer between paired serum samples as the diagnostic criterion, sensitivity of the ST ICT for admission-phase samples was 23.8% (24 of 101; 95% CI = 15.9–33.3%). For patients with an IgM IFA titer ≥ 1:400, sensitivity was 39.1% (147 of 376; 95% CI = 34.1–44.2%). Specificities were 86.2% (126 of 929; 95% CI = 84.1–88.6%) and 99.5% (3 of 654; 95% CI = 98.7–99.9%), respectively. For samples with a ≥ 4-fold increased IFA titer between paired serum samples or IgM IFA titer ≥ 1:400, sensitivity was 34.7% (148 of 426; 95% CI = 30.2–39.5%) and specificity was 99.7% (2 of 604; 95% CI = 98.8–100%) (Table 1).

For the MT IBT, when we used the stringent ≥ 4-fold increase in IFA titer between paired serum samples as the diagnostic criterion, sensitivity of the MT IBT for admission-phase samples was 61.2% (112 of 183; 95% CI = 53.7–68.3%). For patients with an IgM IFA titer ≥ 1:400, sensitivity was 54.6% (185 of 339, 95% CI = 49.1–60.0%). Specificities were moderately high at 86.5% (114 of 847; 95% CI = 84.1–88.8%) and 94.1% (41 of 691; 95% CI = 92.0–95.7%), respectively. For samples with ≥ 4-fold rising IFA titer between paired serum samples or IgM IFA titer ≥ 1:400, sensitivity was 48.4% (198 of 409; 95% CI = 43.5–53.4%) and specificity was 95.5% (28 of 621; 95% CI = 93.5–97.0%) (Table 1).

2) What is the relative accuracy of the admission ST ICT and MT IBT for the detection of IgM compared with the gold standard *O. tsutsugamushi* and *R. typhi* admission-phase IgM IFA assays?

**Figure 1.**
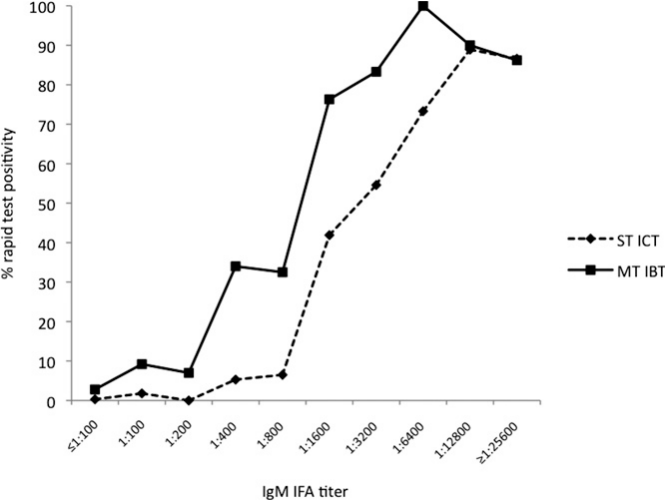
Proportional positivity for the scrub typhus immunochromatograpic test and the murine typhus immunoblot test for detection of IgM compared with the gold standard IgM indirect immunofluorescent antibody assay.

There was a significant association between ST ICT and MT IBT positivity rates and increasing IFA IgM titers (ST ICT and MT IBT Pearson's χ^2^ = 697.2 and 445.4, respectively; both *P* < 0.00005) (Figure 1). The MT IBT was more sensitive for detecting antibodies to *R. typhi* than the ST ICT was for detecting antibodies to *O. tsutsgamushi*, and the lowest IFA titers to reach the 50% sensitivity threshold were 1:1,600 and 1:3,200, respectively (Figure 1). Importantly, the diagnostically informative > 80% sensitivity threshold was reached at IFA titers of 1:12,800 for the ST ICT and 1:3,200 for the MT IBT (Figure 1). This finding was also clearly demonstrated by increased accuracy when ST ICT and MT IBT results for paired samples with admission IgM titers ≥ 1:3,200 were compared. In the case of the ST ICT (n = 147), sensitivity for antibody to *O. tsutsugamushi* was 83.0% (95% CI = 75.9–88.7%) and the specificity was 96.8% (95% CI = 95.4–97.9%). For the MT IBT (n = 74) sensitivity for antibody to *R. typhi* was 94.6% (95% CI = 86.7–98.5%) and specificity was 83.7% (81.2–86.0%).

3) What is the effect of cross-reactivity between antibodies to *O. tsutsugamushi* and *R. typhi* on the diagnostic utility of ST ICT and MT IBT compared with gold standard IFA assays for antibodies against *O. tsutsugamushi* and *R. typhi*?

We investigated the utility of the ST ICT and MT IBT to detect heterologous antibodies for detection of both typhus diseases. Cross-reactivity in the MT IBT for detection of antibodies to scrub typhus ranged from 16.7% to 17.8% depending on the diagnostic criteria used (Table 2). When we used the stringent four-fold increasing diagnostic criteria, of 101 samples indicating scrub typhus, 17.8% (95% CI = 11.6–26.4%) showed a positive reaction in the MT IBT. The ST ICT was marginally more specific than the MT IBT; murine typhus antibody cross-reactivity ranging from 9.8% to 15.9% depending on the diagnostic criteria used (Table 2). When we used the stringent four-fold increasing diagnostic criteria, of 183 samples indicating murine typhus, 9.8% (95% CI = 6.3–15.1%) showed a positive reaction in the ST ICT. There was no significant difference in cross-reactivity between the two assays when the diagnostic criteria were used.

4) What is the effect of sample timing, in relation to the duration of illness, on the ST ICT and MT IBT positivity rates for IgM against *O. tsutsugamushi* and *R. typhi* IFA assay-positive patients?

**Figure 2.**
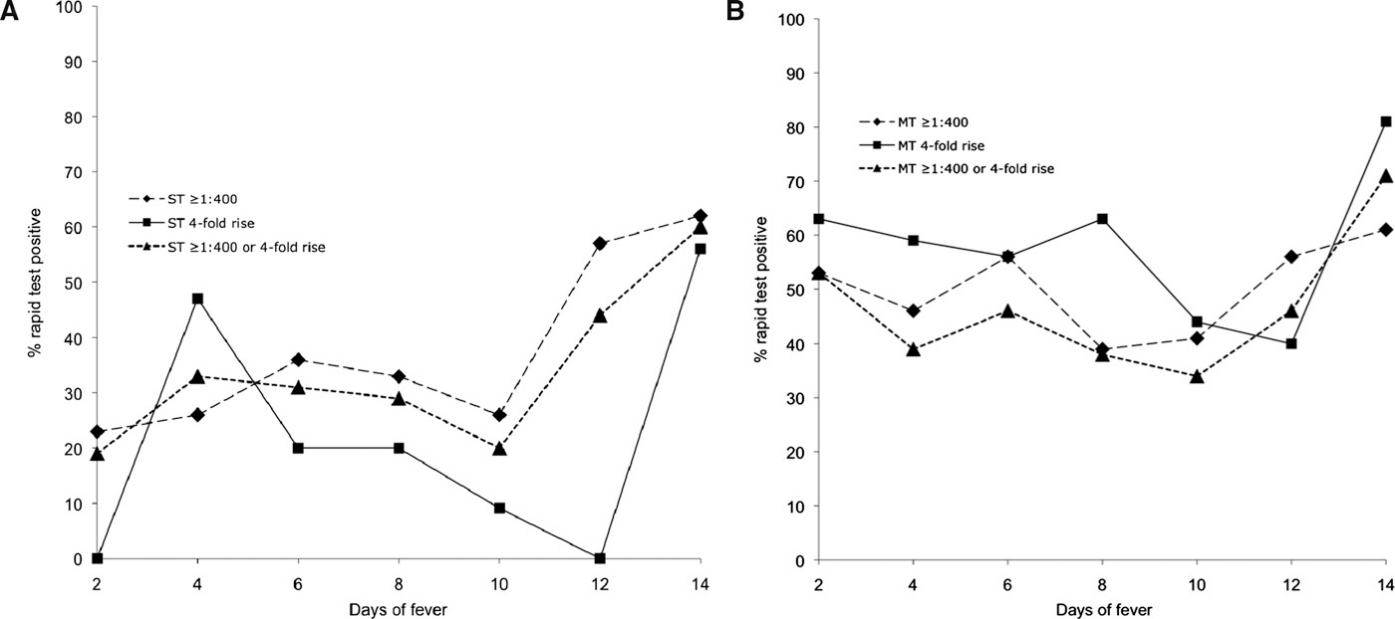
**A**, Effect of sample collection timing (days of illness) on positivity of the immunochromatographic test for detection of IgM against scrub typhus compared with reference assay positivity criteria of a scrub typhus IgM titer ≥ 1:400 in the acute-phase sample, a ≥ 4-fold increase in IgM in paired sample collections, and a combined ≥ 4-fold increase in IgM and a ≥ 1:400 IgM titer result. **B**, Effect of sample collection timing (days of illness) on positivity of the immunoblot test for the detection of IgM against murine typhus compared with reference assay positivity criteria of murine typhus IgM titer ≥ 1:400 in the acute-phase sample, a ≥ 4-fold increase in IgM in paired sample collections, and a combined ≥ 4-fold increase in IgM and ≥ 1:400 IgM titer result.

Rapid test positivity rates for the three reference diagnostic criteria were largely constant with increasing days of illness for both rapid tests until day 10 or 12 of fever when there was a marked increase in positivity (Figure 2). The ST ICT positivity rates remained at < 50% from day 1 to day 10 of illness when there was an increase to approximately 60% positivity (Figure 2A). The MT IBT maintained a positivity range of 40–60% from day 1 to day 12, followed by an increase to approximately between 60% and 80% positivity (Figure 2B).

## Discussion

This study examined the accuracy of the Panbio scrub typhus ICT and the murine typhus IBT in IgM formats for diagnosis of scrub and murine typhus in a prospectively recruited series of patients with typhus-like illness in an area where scrub and murine typhus are common.^2^

The ST ICT and MT IBT had low sensitivity for accurate diagnosis of typhus for admission-phase specimens, which suggested that these rapid assays have limited usefulness for diagnosis of typhus-like illness in this disease-endemic setting. Comparison with reference IFA techniques demonstrated that much higher antibody titers than patients would normally have on admission (range = 1:3,200–12,800) were required to generate positive results in the rapid assays. Sensitivity of these assays may be improved by increasing the amount of antigen on the test strips.

The time when the sample was obtained was an important factor in accuracy of the rapid tests. The ST ICT did not show acceptable levels of sensitivity until well after the median day of presentation in this study (seven days), which was reflected in poor overall sensitivity results. This effect was less pronounced for the MT IBT.

Given that the current recommendations for treatment of scrub and murine typhus are similar, oral doxycycline for seven days,^8^ a test that would be reactive for scrub and murine typhus would be potentially useful. In this study, we investigated the applications of the MT IBT for this purpose because it demonstrated cross-reactivity for patients with scrub typhus at a level that approached that of the ST ICT. However, the sensitivity was insufficient for application with admission-phase samples.

For the scrub typhus ICT, accuracy estimates were lower than those in previous studies with the same product; sensitivities ranged from 74% to 96% and specificities ranged from 86% to 99%.^9^,^10^ Previous studies have evaluated the murine typhus IBT for detection of total antibodies rather than only IgM.^6^,^11^,^12^ Sensitivity and specificity ranged from 51.4% to 100%, respectively, for prospectively collected samples^12^ to 91.4% and 87.7%, respectively, for stored serum samples.^11^ Results in this study are similar to those of previous studies although accuracies were generally lower.

In this study, we used three diagnostic reference standards; the four-fold increasing antibody titer in paired samples, an admission IFA IgM titer ≥ 1:400,^7^ and a combination of both criteria. The application of these different diagnostic criteria demonstrated clear differences in sensitivity results. The IFA IgM titer ≥ 1:400 diagnostic criterion showed lower sensitivity and positivity for the MT IBT than the supposedly more stringent four-fold increasing antibody titer in paired samples. A clear discrepancy was also noted between the two reference standards when ST ICT positivity was compared with increasing time of sample collection; this was less of an the issue with the MT IBT. Specificity results were much lower for both tests when the four-fold increasing antibody titer in paired samples was used as the diagnostic criterion. This result reinforces the problem with application of an appropriate IFA assay diagnostic criteria and/or reference standard for rickettsial diseases^13^ which is exacerbated by residual IgM against scrub typhus or murine typhus from previous infections, which may persist for many months.^7^

An additional issue affecting sensitivity is that it is likely that *O. tsutsugamushi* in Laos are antigenically diverse,^14^ and there may be strains undetected by antibodies against the Karp, Kato, and Gilliam strains. Similar issues may affect *R. typhi* diagnosis, but there are few data to make this judgment.

This study has demonstrated that in a low-resource, typhus-endemic setting, the Panbio scrub typhus ICT and murine typhus Dip-S-Ticks IBT for detecting IgM have potential for diagnosing acute infections. With a high specificity a positive result in either test is a reliable marker of scrub typhus or murine typhus but, in their current format, they have low sensitivity to be clinically useful. Further development of these tests, perhaps with a lower antibody cut-off value, as expressed visually, may increase the sensitivity.

## Figures and Tables

**Table 1:** Sensitivity and specificity for the scrub typhus ICT and murine typhus IBT for detection of IgM compared with the gold standard IgM antibody IFA assay*

Admission sample gold standard IFA diagnostic criteria	Scrub typhus IgM ICT			Murine typhus IgM IBT		
	No. (n = 1,030)	Sensitivity, % (95% CI)	Specificity, % (95% CI)	No. (n = 1,030)	Sensitivity, % (95% CI)	Specificity, % (95% CI)
Four-fold increase in IgM (paired samples)	101	23.8 (15.9–33.3)	86.2 (84.1–88.6)	183	61.2 (53.7–68.3)	86.5 (84.1–88.8)
IgM ≥ 1:400 on admission	376	39.1 (34.1–44.2)	99.5 (98.7–99.9)	339	54.6 (49.1–60.0)	94.1 (92.0–95.7)
Four-fold increase in IgM or IgM ≥ 1:400 on admission	426	34.7 (30.2–39.5)	99.7 (98.8–100)	409	48.4 (43.5–53.4)	95.5 (93.5–97.0)

* ICT = immunochromatographic test; IBT = immunoblot test; IFA = indirect immunofluorescent antibody; CI = confidence interval.

**Table 2:** Proportional heterologous reactivity of scrub typhus ICT and murine typhus IBT for detection of IgM compared with differing gold standard IgM IFA assay diagnostic criteria*

Diagnostic criteria	% Heterologous cross reaction (95% CI)				χ^2^ (*P*)
	No.	Scrub typhus–positive samples with a positive result in murine typhus IgM IBT	No.	Murine typhus–positive samples with a positive result in scrub typhus IgM ICT	
Four-fold increase in IgM	101	17.8 (11.6–26.4)	183	9.8 (6.3–15.1)	2.85 (0.09)
IgM ≥ 1:400 admission	376	17.6 (14.0–21.7)	339	15.0 (11.6–19.2)	0.66 (0.80)
Four-fold increase in IgM or IgM ≥ 1:400 on admission	426	16.7 (13.4–20.5)	409	15.9 (12.7–19.8)	0.59 (0.44)

* ICT = immunochromatographic test; IBT = immunoblot test; IFA = indirect immunofluorescent antibody; CI = confidence interval.
