# Erratum

**DOI:** 10.4269/ajtmh.2010.834err

**Published:** 2010-10-05

In *Am J Trop Med Hyg 83:* 365–369 by Blacksell et al., there is an error in the second to last sentence on page 369. The sentence should read “With a high specificity a positive result in either test is a reliable marker of scrub typhus or murine typhus but, in their current format, they have low sensitivity.” The journal regrets this error.

